# Clickable Dynamic Bioinks Enable Post‐Printing Modifications of Construct Composition and Mechanical Properties Controlled over Time and Space

**DOI:** 10.1002/advs.202300055

**Published:** 2023-09-15

**Authors:** Pierre Tournier, Garance Saint‐Pé, Nathan Lagneau, François Loll, Boris Halgand, Arnaud Tessier, Jérôme Guicheux, Catherine Le Visage, Vianney Delplace

**Affiliations:** ^1^ RMeS – Regenerative Medicine and Skeleton (INSERM UMR 1229) Oniris, CHU Nantes, INSERM Nantes Université Nantes F‐44000 France; ^2^ Laboratoire CEISAM (UMR CNRS 6230) Nantes Université Nantes F‐44000 France

**Keywords:** bioinks, bioprinting, click chemistry, dynamic hydrogel, hyaluronic acid

## Abstract

Bioprinting is a booming technology, with numerous applications in tissue engineering and regenerative medicine. However, most biomaterials designed for bioprinting depend on the use of sacrificial baths and/or non‐physiological stimuli. Printable biomaterials also often lack tunability in terms of their composition and mechanical properties. To address these challenges, the authors introduce a new biomaterial concept that they have termed “clickable dynamic bioinks”. These bioinks use dynamic hydrogels that can be printed, as well as chemically modified via click reactions to fine‐tune the physical and biochemical properties of printed objects after printing. Specifically, using hyaluronic acid (HA) as a polymer of interest, the authors investigate the use of a boronate ester‐based crosslinking reaction to produce dynamic hydrogels that are printable and cytocompatible, allowing for bioprinting. The resulting dynamic bioinks are chemically modified with bioorthogonal click moieties to allow for a variety of post‐printing modifications with molecules carrying the complementary click function. As proofs of concept, the authors perform various post‐printing modifications, including adjusting polymer composition (e.g., HA, chondroitin sulfate, and gelatin) and stiffness, and promoting cell adhesion via adhesive peptide immobilization (i.e., RGD peptide). The results also demonstrate that these modifications can be controlled over time and space, paving the way for 4D bioprinting applications.

## Graphical abstract

This article introduces a new biomaterial concept termed “clickable dynamic bioinks”. These bioinks use dynamic hydrogels that can be 3D‐bioprinted and chemically modified via click and bioorthogonal reactions to allow a variety of post‐printing modifications. These modifications include adjusting the bioink composition and stiffness, as well as promoting cell adhesion, and can be controlled over time and space for 4D bioprinting applications.

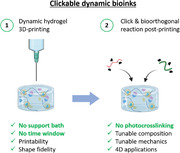

## 1 Introduction

3D bioprinting technology holds great promise toward advancing the fields of tissue engineering and regenerative medicine,^[^ ^1^ ^]^ with applications spanning from disease modeling to drug testing and tissue replacement.^[^ ^2^, ^3^, ^4^, ^5^ ^]^ Bioprinting allows for the 3D arrangement of biomaterials and cells, commonly coupling a printing technology such as extrusion, inkjet, or volumetric bioprinting, with a class of printable materials obtained from natural (i.e., alginate, gelatin, collagen, or hyaluronic acid [HA]) or synthetic (i.e., polyethylene glycol [PEG]) polymers.^[^ ^1^, ^6^, ^7^, ^8^ ^]^ Extrusion bioprinting is the most commonly used bioprinting technology, likely because it can produce objects the size of natural tissues within minutes using relatively affordable machines and conventional extrudable biomaterials (e.g., alginate and/or gelatin). Recent developments in 3D printing and bioprinting technologies have facilitated multimaterial 3D printing,^[^ ^9^ ^]^ higher printing resolutions (in the order of 20 µm),^[^ ^10^ ^]^ as well as impressively fast 3D printing (sometimes below 30 s for centimeter scale chess pieces).^[^ ^11^, ^12^ ^]^ However, available printable materials, also named biomaterial inks, are most often hydrogels containing polymers with inherent gelation properties that do not allow to independently tune their composition and mechanical properties. This limits our ability to recapitulate simultaneously the physical and biochemical characteristics of natural extracellular matrices (ECM), which is required to design biologically relevant constructs.^[^ ^7^, ^13^, ^14^ ^]^ The ideal biomaterial ink for extrusion bioprinting would prevent cell sedimentation in the cartridge; enable the 3D printing of cells without affecting their viability and biological functions; offer a straightforward printing process, ideally without the need for a support bath or a specific printing setup (e.g., light‐based bioprinter), and/or an additional chemical reaction; produce constructs with both good shape fidelity and stability in culture medium under physiological conditions of salt, pH, and temperature; and be tunable in terms of composition and mechanical properties.

With the emergence of 4D printing,^[^ ^14^, ^15^, ^16^, ^17^, ^18^ ^]^ the ability to control the composition and mechanical properties of a construct in time and space is also becoming desirable. This would be particularly relevant for reproducing, in vitro, aging and disease development where matrix alterations are commonly observed (e.g., fibrotic processes, tissue degeneration). However designing materials for 4D bioprinting remains challenging. One key challenge is having fine‐tuned control over the physical transition of the printable biomaterial from a liquid to solid state (i.e., the sol‐gel transition) in the presence of cells, while also being able to tune its final physical and biochemical properties. Hydrogels, which are hydrated polymer networks, can recapitulate many ECM characteristics (i.e., hydration, composition, and mechanics) and are thus widely considered to be the best candidates to address the criteria for 4D bioprinting. However, to date, most printable hydrogels have relied on the use of conventional polymers (e.g., gelatin, alginate, and collagen) that spontaneously form hydrogels under non‐physiological stimuli (i.e., non‐physiological temperature, pH, or salt concentration), resulting in biomaterial inks with inherently limited properties. Typically, gelatin forms a hydrogel upon cooling and dissolves at a physiological temperature of 37 °C, meaning it can only be used as a sacrificial material;^[^ ^9^ ^]^ similarly, alginate crosslinking requires a non‐physiological calcium concentration of >1.8 mM, which can induce cell apoptosis;^[^ ^19^ ^]^ and collagen only forms a hydrogel upon a transition from an acidic to neutral pH (typically, from pH = 3.5 to 7.4), making cell encapsulation difficult.^[^ ^20^ ^]^

To address the limitations associated with the sol‐gel transition, as well as the common issue of structure collapse associated with conventional layer‐by‐layer printing, innovative support baths have been developed. For example, in their pioneering study, Feinberg et al used a support bath composed of gelatin microbeads to print acellular viscous solutions in suspension with a high printing resolution (down to 20 µm) prior to in situ crosslinking.^[^ ^20^, ^21^, ^22^ ^]^ Among others, this strategy was used to print HA hydrogels with additional peptide‐mediated supramolecular interactions controlled over time.^[^ ^23^ ^]^ The technology was later associated with a new in situ chemical crosslinking based on the diffusion of a PEG crosslinker from the bath toward the printed filament, allowing the researchers to incorporate cells and tune the polymer composition of the construct.^[^ ^24^ ^]^ Although promising, this strategy still depends on using a specific support bath and requires incubating the construct for several hours under non‐physiological conditions, such as at 4 or 20 °C, to complete the crosslinking process, making it relatively difficult to implement. As alternatives to using gelatin as a sacrificial material, a few preliminary studies have demonstrated the feasibility of printing noncovalent, supramolecular or dynamic covalent hydrogels, which are viscoelastic and can easily flow, before rigidifying them via covalent crosslinking.^[^ ^25^, ^26^, ^27^ ^]^ However, all of these systems used UV photo‐crosslinking for the post‐printing rigidifying step, which is a process that both requires a specific printing setup and is potentially harmful to the cells. In general, all attempts to combine printability and tunability have so far required either a multistep printing process or non‐physiological conditions, as well as a support bath, a sacrificial material, and/or photo‐crosslinking.

Here, we hypothesized that a dynamic covalent hydrogel that can be 3D printed in the presence of cells can be modified with reactive moieties. The mechanical and biochemical properties of these printed objects can then be adjusted after printing simply by adding a complementary reactive molecule to the culture medium. Using HA as a common polymer of interest, we first investigated the use of boronate ester‐based dynamic covalent crosslinking for the design of dynamic bioinks. To date, boronate ester‐based bioink design has been the focus of only two articles, both presenting hydrogels composed of alginate and calcium for co‐ionic crosslinking, with limited tunability.^[^ ^28^, ^29^ ^]^ Thus, we set out to develop a new boronate ester‐based system. We took advantage of a universal boronate ester‐based crosslinking strategy (i.e., reaction between Wulff‐type phenylboronic acid [wPBA] and glucamine) recently developed by our group to produce dynamic hydrogels with viscoelastic properties.^[^ ^30^ ^]^ We previously showed that carefully adjusting the HA molecular weight, degrees of substitution, polymer content, and wPBA:glucamine molar ratio allows for the design of viscoelastic hydrogels that are noswelling and stable long term under physiological conditions. We evaluated the cytocompatibility of the optimal dynamic hydrogels and the associated printing conditions using primary human adipose‐derived stromal cells and chondrocytes, as well as evaluated whether using dynamic hydrogels can avoid cell sedimentation in the bioprinting cartridge, which together would confirm the successful design of dynamic bioinks. Regarding the second reaction, we investigated the use of strain‐promoted azide‐alkyne cycloaddition (SPAAC)^[^ ^24^ ^]^ as a click and bioorthogonal reaction^[^ ^5^ ^]^ able to meet all of the criteria for post‐bioprinting modifications, including feasibility under physiological pH and temperature, and the absence of byproducts.^[^ ^31^ ^]^

**Figure 1:**
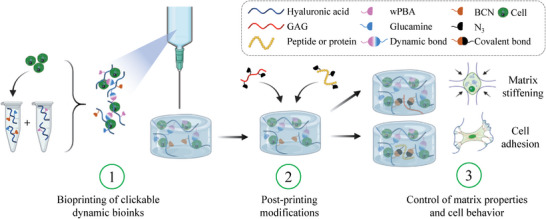
General concept of clickable dynamic bioinks. A boronate ester‐based dynamic covalent hydrogel, which can be printed in the presence of cells, is chemically modified to allow post‐printing modifications of the composition and mechanical properties of printed constructs via a bioorthogonal and click reaction (i.e., SPAAC). Possible post‐printing modifications include adjusting polymer composition and stiffness, as well as promoting cell adhesion, all of which can be controlled in time and space, facilitating 4D bioprinting applications.

To demonstrate the potential of the proposed biomaterials, which we termed “clickable dynamic bioinks” (**Figure** **1**), we performed a variety of post‐printing modifications, including adjusting the polymer composition (e.g., HA, chondroitin sulfate [CS], etc.) and stiffness, and promoting cell adhesion with adhesive macromolecules (e.g., gelatin and RGD peptide). Of major interest, we showed that these post‐printing modifications can be performed at different times (at least over 7 days) and controlled in space, laying the foundation for 4D bioprinting applications.

## 2 Results and Discussion

### 2.1 Boronate Ester‐Based Hydrogels as Novel 3D Printable Biomaterials

**Figure 2:**
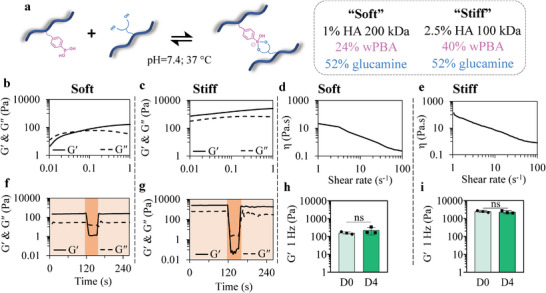
Rheological behavior of optimal boronate ester‐based hydrogel formulations for 3D bioprinting. a) Two formulations were selected for their minimal swelling and distinct mechanical properties, and called “soft” and “stiff”, respectively. b–i) The rheological characterization of the soft (b,f,d,h) and stiff (c,g,e,i) dynamic hydrogels. b,c) Frequency‐dependent storage modulus (Gʹ) and loss modulus (G″) of the two formulations, confirming the dynamic nature of the hydrogels. d,e) Shear‐thinning behavior of the proposed hydrogels, characterized by a decrease in viscosity when increasing shear rate. f,g) Demonstration of the self‐healing properties of the hydrogels. Light and dark orange overlays indicate periods of low stress (1 Pa) and high stress (500 Pa for “soft”; 1000 Pa for “stiff”), respectively. h,i) Storage moduli of the hydrogels on day 0 (D0) and after 4 days (D4) in PBS, confirming the mechanical stability of the hydrogels. Student's t‐tests were performed and statistical differences set at *p* < 0.05.

To design clickable dynamic bioinks, we first investigated the use of boronate ester‐based hydrogels as printable biomaterials for extrusion bioprinting. HA was modified with either wPBA or glucamine via an amidation procedure under aqueous conditions using DMT‐MM as a coupling agent (Table S1, Supporting Information). Boronate ester‐based hydrogels were obtained upon mixing HA‐wPBA and HA‐glucamine solutions. Hydrogel formulations were optimized to produce printable hydrogels with distinct mechanical properties (Gʹ, at 1 Hz and at 37 °C of ≈200 Pa and ≈2000 Pa for the formulations be hereafter referred to as “soft” and “stiff”, respectively) (**Figure** **2**a) and satisfactory post‐printing stability, i.e., self‐standing capacity and lack of swelling. After systematically screening various polymer contents (1% versus 2.5% [w/v]), HA molecular weights (100 versus 200 kDa), and degrees of substitution (DS) of HA (wPBA DS of 24% versus 40%), two printable formulations (Table S2, Supporting Information) with distinct mechanical properties (Figure 2) were selected for their minimal swelling (Figure S1, Supporting Information) with the aim to prevent shape alteration upon construct immersion in culture medium.

Our investigation of the rheological properties of the two formulations indicated that both dynamic hydrogels formed within seconds upon the simple mixing of the two HA precursor solutions and reached their chemical equilibria within 30 min and 120 min for the soft and stiff hydrogel, respectively (Figure S2, Supporting Information). As expected, both formulations exhibited frequency‐dependent storage (Gʹ) and loss (G″) moduli typical of dynamic covalent network formation (Figure 2b,c). The stiff formulation maintained a solid‐like behavior over a larger range of shear rates, suggesting that the molecular weight, polymer content and crosslinking density play a role in the rheological properties of HA‐based boronate ester hydrogels, as previously reported.^[^ ^30^ ^]^ We further confirmed the shear‐thinning behavior of the two hydrogel formulations with shear rate ramp experiments (Figure 2d,e). To validate the use of these dynamic hydrogels for bioprinting, we then evaluated their ability to flow upon stress and to recover their mechanical properties using alternated stress sweep tests (alternating 1 and 1000 Pa). Following network disruption under temporary high stress, which was indicated by a drop in Gʹ values below G″ values, both hydrogels were able to instantaneously recover their initial shear moduli, confirming their self‐healing properties (Figure 2f,g). We then investigated the maintenance of the mechanical properties of the two hydrogels over time—a major criterion for bioprinting applications. Both formulations showed no significant change in their Gʹ values after 4 days of immersion in PBS at 37 °C, confirming their stability under physiological conditions (Figure 2h,i).

**Figure 3:**
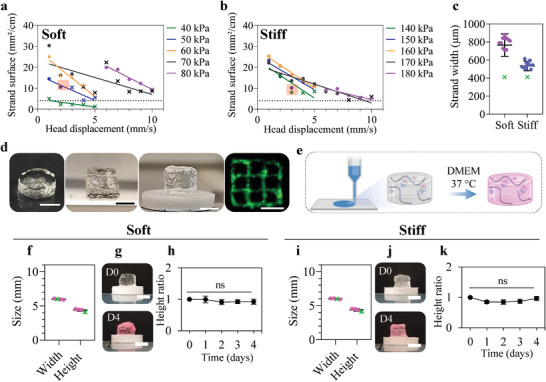
Printability and self‐standing capacity of boronate ester‐based hydrogels. a,b) Printed 1 cm‐long strand surface as a function of input air pressure and print head displacement speed using a 22‐gauge needle for extrusion. Light red squares indicate the area of optimal printing parameters corresponding to minimal strand surfaces. Dots indicate continuous printed strands, while crosses indicate fragmented strands. The dashed lines indicate the value of the ideal strand surface (4.1 mm^2^) associated with extrusion through a 22‐gauge needle. c) The width of strands generated with the optimal printing parameters determined for each formulation. The green crosses indicate the inner diameter of the needle as a reference. d) Illustrations of centimeter‐sized objects of various shapes, including cylinders, cubes, and grids (scale bars: 5 mm), obtained by using the soft formulation. e) Schematic of the investigation of boronate ester‐based hydrogel self‐standing capacity: boronate ester‐based hydrogels were prepared with PBS and 3D printed in a filled cylindrical shape, then immersed in culture medium for 4 days at 37 °C to evaluate shape maintenance. f,i) Evaluation of the 3D printability of filled cylinders designed with the soft and stiff hydrogels. The obtained cylinders had dimensions comparable to the expected values (indicated as green crosses). g,j) Pictures of the printed cylinders on day 0 and after 4 days of immersion, showing minimal changes in dimensions (scale bars: 2.5 mm). h,k) The height ratio (normalized to day 0) of the 3D printed cylinders monitored over 4 days, demonstrating that the dynamic hydrogels did not collapse. Statistical significance was evaluated using a one‐way ANOVA with Tukey's post‐hoc test (ns = not significant).

After demonstrating their suitable rheological properties for bioprinting, we evaluated the printability of the soft and stiff hydrogels in 2D and 3D using 22‐gauge needles. We determined their optimal printing parameters by systematically screening combinations of input air pressures of up to 80 and 180 kPa for the soft and stiff formulations, respectively, and printing head displacement speeds of 1 to 10 mm.s^−1^ (**Figure** **3**a,b). The surface of printed strands (1 cm long) was measured to identify the printing conditions that provided the thinnest strands without strand discontinuity. As expected, both increasing the air pressure and decreasing the speed of head displacement led to thicker strands, which is associated with decreased resolution. Conversely, high speeds without sufficient pressure resulted in strand discontinuity. Overall, we observed linear relationships between pressure and head displacement. Based on these findings, we selected the optimal printing parameters for each formulation as follows: an air pressure of 51 kPa and head displacement of 1.5 mm.s^−1^ for the soft hydrogel, and an air pressure of 152 kPa and head displacement of 3 mm.s^−1^ for the stiff hydrogel. We successfully printed 2D grids (Video S1 and S2, Supporting Information) and confirmed that the process was reproducible by measuring strand width (Figure 3c). Compared to the soft formulation, the higher viscosity and mechanical properties of the stiff formulation led to less spreading and allowed us to generate strands with widths (600 to 800 µm) closer to the inner diameter (410 µm) of the 22‐gauge needle. However, while the stiff formulation could not be printed with thinner needles, the soft hydrogel could, where a 27‐gauge needle with an inner diameter of 210 µm typically led to thinner strands (down to ≈ 200 µm). In sum, the optimized boronate ester‐based hydrogels presented here using wPBA and glucamine as crosslinking moieties on HA polymers are printable under conditions compatible with 3D bioprinting.

We then investigated whether the two dynamic hydrogels are suited to 3D printing, as well as the post‐printing stability of their resulting 3D constructs. Using the aforementioned printing parameters, we generated centimeter‐scale constructs of various shapes (i.e., cylinder, square) up to 10‐layers in height (Figure 3d; soft formulation). Focusing on 3D cylinders (6 mm wide with 10 layers), we showed that 3D printing with both formulations produced objects with the expected height and width (Figure 3i,j). Despite their flowing properties, we found that our hydrogels were elastic enough to allow tens of minutes of printing before construct collapse. Such phenomenon eventually occurs (after ≈15 min and ≈1 h for the soft and stiff hydrogels, respectively) when the hydrogels are exposed to the air for too long, which is due to the surface tension at the hydrogel‐air interface in combination with the dynamic nature of the networks. The risk of collapse is completely alleviated by immersing the printed constructs in medium, which we believe is due to the quasi‐absence of surface tension at the hydrogel‐medium interface. Indeed, we observed no significant variation in height for 3D‐printed cylinders immersed in culture medium at 37 °C for 4 days, demonstrating the self‐standing capacity and in vitro stability of the printable hydrogels (Figure 3g,h,j,k). While other dynamic covalent hydrogels have reportedly been used for bioprinting,^[^ ^9^, ^24^, ^32^ ^]^ to our knowledge, our proposed boronate ester‐based hydrogels are the first dynamic covalent hydrogels to combine printability and stability under physiological conditions without the need for a support bath or subsequent crosslinking. However, it is worth noting that the dynamic nature of our hydrogels makes them prone to coalescence, limiting their use for the design of 3D printed constructs with high resolution or hollow parts. In general, boronate ester‐based hydrogels can be used to print 3D objects that are viscoelastic, which could potentially better mimic the mechanical features of ECMs.^[^ ^33^, ^34^, ^35^, ^36^, ^37^ ^]^

### 2.2 Boronate Ester‐Based Hydrogels for Bioink Design

**Figure 4:**
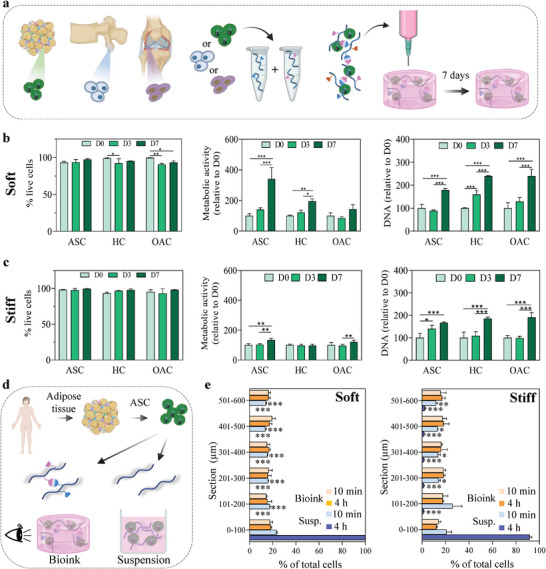
Boronate ester‐based hydrogels for dynamic bioink design. a) Schematic of the evaluation of the cytocompatibility of the printing process associated with boronate ester‐based hydrogels using three cell types: human adipose‐derived stromal cells (ASCs), chondrocytes from healthy vertebrae (HCs), and chondrocytes from osteoarthritic knees (OACs). b,c) Evaluation of the cytocompatibility of soft and stiff boronate ester‐based hydrogels in combination with the printing process over 7 days, including the assessment of cell viability, cell metabolic activity, and DNA content. d,e) Evaluation of cell sedimentation in soft and stiff dynamic bioinks compared to HA solutions, demonstrating the absence of cell sedimentation only when using dynamic hydrogels. Data are shown as mean ± SD (N = 3 distinct polymer batches) with statistical significance determined by two‐way ANOVA with Tukey's post‐hoc test (*: *p*<0.05; **: *p*<0.01; ***: *p*<0.001).

Next, we evaluated the possible use of these boronate ester‐based hydrogels for designing bioinks. Recently, we demonstrated the cytocompatibility of the new boronate ester‐based hydrogels using a model cell line of murine fibroblasts (L929) and primary human adipose‐derived stromal cells (ASCs).^[^ ^30^ ^]^ However, confirming the cytocompatibility of the hydrogels in combination with the bioprinting process is crucial. Thus, we investigated the viability, metabolic activity, and proliferation of various cell types after encapsulation and bioprinting using ASCs, chondrocytes from healthy vertebrae (HCs), and chondrocytes from osteoarthritic knees (OACs) (**Figure** **4**a). Each cell type was independently encapsulated in either soft or stiff hydrogels and extruded with a bioprinter in multiwell plates prior to being evaluated. Over 7 days of culture, our data showed high cell viability (≥ 85%), irrespective of the cell type and hydrogel formulation used (Figure 4b,c; Figure S3, Supporting Information). Regarding cell metabolism, interestingly, our data revealed that cells encapsulated in the stiff formulation exhibited lower increase to no increase in their metabolic activity over time compared to cells encapsulated in the soft formulation. This may be attributed to the higher mechanical properties of the stiff hydrogel (i.e., higher storage and compressive moduli) compared to those of the soft hydrogel, which may increase cell confinement and affect cellular functions in 3D.^[^ ^38^, ^39^ ^]^ Finally, by assessing the DNA content, which is directly related to the number of cells, we showed that the DNA content systematically increased over time, suggesting cell proliferation within the dynamic hydrogels.

Because boronate ester‐based hydrogels can flow and thus be loaded into cartridges after crosslinking, we hypothesized that they could prevent cell sedimentation and printing time constraints, which are major challenges in 3D bioprinting.^[^ ^40^, ^41^ ^]^ Using ASCs as model cells, we evaluated their sedimentation over time after encapsulation in dynamic hydrogels (Figure 4d). As controls, cells were resuspended in solutions of unmodified HA with polymer molecular weights and concentrations similar to those of the soft and stiff hydrogels (1% [w/v] 200 kDa and 2.5% [w/v] 100 kDa, respectively). These controls led to complete cell sedimentation within hours (Figure 4e; Figure S4, Supporting Information). In contrast, cells remained homogeneously distributed in both soft and stiff hydrogels, with no significant difference in cell number per hydrogel section between 10 min and 4 h, confirming that dynamic hydrogels circumvent the common issue of cell sedimentation. Together, our data demonstrated the cytocompatibility of both dynamic hydrogel formulations when combined with the extrusion bioprinting process, validating the use of these hydrogels for bioink design.

### 2.3 Clickable Dynamic Bioinks

#### 2.3.1 Proof of Concept

The main challenge in designing bioinks is combining printability with complete control over the composition (i.e., polymer and/or peptide) and mechanical properties (e.g., stiffness viscoelasticity) of bioprinted constructs. To overcome this challenge, we modified our newly‐designed dynamic bioinks with clickable moieties to enable a variety of post‐printing modifications. As a second chemical reaction platform, we selected the SPAAC reaction between bicyclononyne (BCN) and azide (N_3_), which is a bioorthogonal and click reaction^[^ ^24^, ^42^ ^]^ that uses off‐the‐shelf compounds. To obtain clickable dynamic bioinks, the HA‐glucamine precursor for our dynamic hydrogels was modified with BCN (Figure S5 and Table S1, Supporting Information) before dynamic hydrogel formation, producing BCN‐modified dynamic hydrogels (Table S2, Supporting Information) that are able to react with diffusible N_3_‐bearing molecules after adding them to the culture medium.

**Figure 5:**
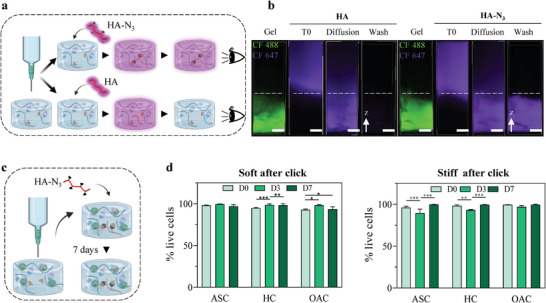
Clickable dynamic bioinks. a) Schematic of the general strategy to adjust the composition of the dynamic bioinks after printing: a 3D‐printed clickable dynamic hydrogel is immersed in PBS containing either unmodified HA (20 kDa) or HA‐N_3_ (20 kDa). b) Projections of confocal images of extrusion‐printed clickable dynamic hydrogels (labeled with CF 488), immersed in CF 647 HA (top left) or CF 647 HA‐N_3_ (top right), with respective fluorescence quantification along the z‐axis (bottom left and right). The dashed lines indicate the upper limit of the hydrogels (scale bars: 500 µm). c) Schematic of the evaluation method of the cytocompatibility of clickable dynamic hydrogels after post‐printing modification with HA‐N_3_: cells are encapsulated in clickable dynamic hydrogels, 3D printed, and immersed in DMEM containing HA‐N_3_ for 24 h before monitoring cell viability over 7 days. d) Evaluation of the cytocompatibility of dynamic hydrogels after the click reaction using ASCs, HCs, and OACs. Data are shown as mean ± SD (N = 3 distinct polymer batches) with statistical significance determined by two‐way ANOVA with Dunnett's post‐hoc test (*: *p*<0.05; **: *p*<0.01; ***: *p*<0.001).

Using rheological measurements, we first confirmed that the BCN modification did not alter the hydrogels’ mechanical properties (Figure S6, Supporting Information). We then evaluated the accessibility of BCN for click reaction with complementary moieties (N_3_‐bearing molecules). To modify BCN‐presenting dynamic hydrogels, the complementary N_3_‐bearing molecule must diffuse through the dynamic network, making molecular diffusion a key element for success. As a proof of concept, we evaluated the successful diffusion and immobilization of fluorescently labeled HA‐N_3_ (20 kDa) compared to that of unmodified HA (20 kDa) (**Figure** **5**a). After 24 h of incubation, both unmodified HA and HA‐N_3_ had successfully diffused throughout the clickable dynamic hydrogels; yet, only the fluorescently labeled HA‐N_3_ remained inside the hydrogel after several washes, demonstrating the covalent and homogeneous immobilization of the N_3_‐modified polymer (Figure 5b; Supplementary Figure S7, Supporting Information). A similar procedure showed that the same HA‐N_3_ was not immobilized in a non‐clickable dynamic hydrogel (i.e., without BCN modification), further confirming the specificity of the second click reaction (Figure S8, Supporting Information). We also confirmed the absence of swelling or shrinking of the clickable dynamic hydrogels before and after second chemical reaction with diffusible HA‐N_3_ dissolved in culture medium, demonstrating that the second reaction does not alter the stability of the hydrogels (Figure S9, Supporting Information). Theoretically, using this strategy to modify the composition post‐printing can be achieved with any molecule modified with N_3_ groups, as long as they can diffuse throughout the construct. Next, we verified the cytocompatibility of the general process by printing clickable dynamic bioinks before the second click reaction with diffusible HA‐N_3_ in the presence of cells (Figure 5c). After this second click reaction, all cell types tested had a cell viability > 90% at every time point (Figure 5d; Figure S10, Supporting Information), confirming the overall cytocompatibility of our approach.

#### 2.3.2 Post‐Printing Adjustments of Clickable Dynamic Hydrogel Properties

**Figure 6:**
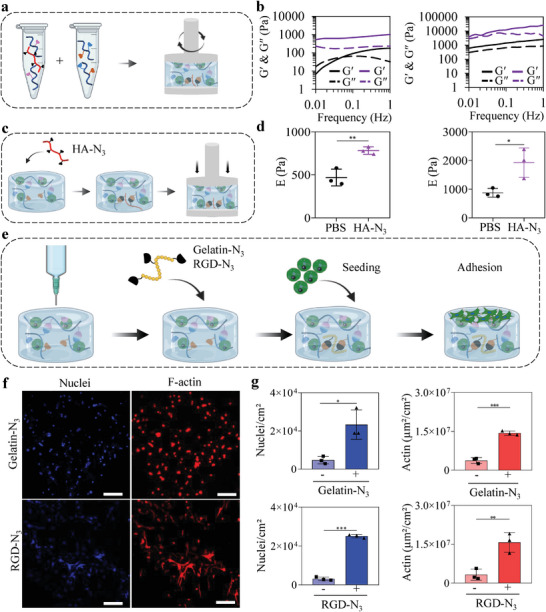
Post‐printing modifications of clickable dynamic bioinks. a) Preliminary experiments on the feasibility of second crosslinking in clickable dynamic bioinks for mechanical reinforcement: HA‐N_3_ (20 kDa) was dissolved with HA‐wPBA before mixing with BCN‐modified HA‐glucamine and rheological assessment. b) Frequency sweep experiments were performed with the soft (left) or stiff (right) clickable dynamic hydrogels in the presence (purple) or absence (black) of HA‐N_3_. c) Mechanical reinforcement experiments on clickable dynamic hydrogels. d) Stiffness increase was evaluated via unconfined compression tests on soft (left) or stiff (right) clickable dynamic hydrogels after 24 h immersion in PBS or HA‐N_3_ (20 kDa). Each data point represents a single hydrogel punched on at least 4 locations. d) Schematic of the experiment on cell adhesion promotion: clickable dynamic hydrogels were immersed in culture medium containing N_3_‐bearing bioactive molecules (gelatin‐N_3_ or RGD‐N_3_) before cell seeding, washes, and cell adhesion evaluation. e,f) Evaluation of ASC adhesion to clickable dynamic hydrogels modified with either gelatin‐N_3_ or RGD‐N_3_ using Hoechst (nuclei, blue) and AF 568 phalloidin (F‐actin, red) staining (scale bars: 200 µm). Each data point represents a different hydrogel. Data are shown as mean ± SD with statistical significance determined by unpaired t‐tests (*: *p* < 0.05; **: *p*<0.01; ***: *p*<0.001).

To explore the potential of clickable dynamic bioinks, we incorporated several N_3_‐modified molecules, including HA, gelatin, and the RGD peptide, with the aim to modify construct rigidity or specific cell‐material interactions (cell adhesion) after printing. First, we investigated how to adjust the mechanical properties of clickable dynamic bioinks via subsequent SPAAC crosslinking. As a preliminary experiment, we evaluated the feasibility of mechanical reinforcement by co‐dissolving HA‐N_3_ (20 kDa) with HA‐wPBA prior to mixing with BCN‐modified HA‐glucamine (N_3_:BCN molar ratio of 1:1) (**Figure** **6**a). One hour after mixing, both time sweep and frequency sweep experiments revealed a 10‐fold increase in the hydrogel shear elastic modulus (Gʹ at 1 Hz) in the presence of HA‐N_3_, compared with the non‐clickable hydrogel control, confirming successful subsequent crosslinking (Figure 6b; Figure S11, Supporting Information). The frequency sweep experiments also showed that the shear elastic moduli of the reinforced hydrogels are frequency independent, further suggesting a transition from a dynamic covalent hydrogel to a predominantly covalent hydrogel. We then investigated the feasibility of mechanical reinforcement via diffusion using clickable dynamic hydrogels immersed in PBS containing HA‐N_3_ (N_3_:BCN molar ratio of 1:1) (Figure 6c). After overnight immersion, unconfined compression tests revealed a 2‐fold increase in Young's modulus (E) compared to the clickable dynamic hydrogel without HA‐N_3_ (Figure 6d) for both the soft and stiff hydrogel formulations. The difference in the level of mechanical reinforcement between the rheological and compressive experiments could suggest that diffusion is less efficient than mixing for second crosslinking. Of note, the applied N_3_:BCN molar ratio of 1:1 is based on the assumption that all of the immobilized N_3_ functions could reach and react with BCN moieties within the hydrogel, which may be debatable. When we tested other molar ratios (1:5 and 5:1), they did not lead to superior mechanical reinforcement (Figure S12, Supporting Information). This could be explained by the fact that, for a given polymer content, a molar ratio of 1:1 theoretically leads to the highest crosslinking density possible, and thus the greatest variation in Young's modulus upon mechanical reinforcement. The limited range of mechanical reinforcement accessible with the current system possibly results from the limited degree of co‐substitution in BCN (i.e., 6%). Overall, these results call for further mechanistic investigations, and alternative click reactions could be tested to expand the range of mechanical properties of our 3D‐printed constructs. Nevertheless, these data confirmed the feasibility of the mechanical reinforcement of a clickable dynamic bioink after printing by simple immersion in a bath containing a diffusible crosslinker.

We then investigated how we could use clickable dynamic bioinks for post‐printing control of cell adhesion via incorporating adhesive motifs. We evaluated the effect of immobilizing two N_3_‐modified molecules with adhesive properties—gelatin (10 mg.mL^−1^) and the RGD peptide (N_3_‐KGSGSGRGDSP, 300 µM)—on the adhesion of ASCs onto clickable dynamic hydrogels (Figure 6e). After overnight incubation, the modified hydrogels were washed several times to eliminate non‐covalently bound adhesive molecules. ASCs were then seeded onto the surface of the hydrogels and left for 24 h. After several washes to remove non‐adhering cells, few cells were found on the top of the hydrogels without immobilized adhesive molecules (Figure S13, Supporting Information). Conversely, incubating clickable dynamic hydrogels with gelatin‐N_3_ or RGD‐N_3_ led to cell adhesion with a significantly higher number of nuclei and actin surfaces compared to controls (Figure 6f,g). Together, our results demonstrated that various post‐printing modifications of clickable dynamic bioinks can be used to control either the physical (e.g., mechanical reinforcement) or biochemical (e.g., adhesion) properties of 3D‐printed constructs.

#### 2.3.3 Controlling Clickable Dynamic Hydrogel Composition Over Time And Space

**Figure 7:**
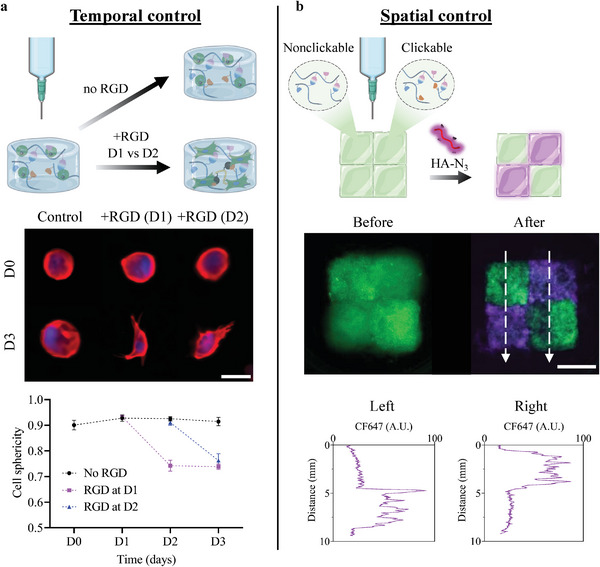
Controlling the post‐printing modification of clickable dynamic hydrogels over time and space. a) Temporal control over cell adhesion using clickable dynamic bioinks. ASCs within 3D bioprinted hydrogels were cultured over 3 days, with or without clickable RGD added to culture medium on day 1 or day 2 (scale bar = 20 µm). Cell sphericity measurements confirmed cell adhesion promotion at different times. Data are shown as mean ± SD (n = 40 cells from 4 different hydrogels). b) Spatial control over macromolecule immobilization. Nonclickable and clickable dynamic hydrogels were printed side‐by‐side, before incubation with CF 647 HA‐N_3_ for 24 h and several washes (scale bar = 5 mm). CF647 fluorescence intensity along the dashed white arrows, confirmed the local modification of clickable dynamic hydrogels.

As clickable dynamic bioinks enable post‐printing modifications of constructs, we finally interrogated whether such post‐printing modifications could be performed independent of time and controlled in space. To test the feasibility of click immobilization at different times post‐printing, we immersed clickable dynamic bioinks for 24 h in PBS containing fluorescently labeled unmodified HA (20 kDa) or HA‐N_3_ (20 kDa) at day 0, 3 or 7 post‐printing. After several washes, no fluorescence was found in the clickable dynamic hydrogels incubated with unmodified HA, while HA‐N_3_ was successfully immobilized in the clickable dynamic hydrogels, irrespective of the time that elapsed before its addition (**Figure** **7**a; Figure S14, Supporting Information). Similar results were obtained for the immobilization of N_3_‐modified CS at different times (Figure S15, Supporting Information), suggesting that this post‐printing modification can be performed with other diffusible N_3_‐modified molecules.

For 4D bioprinting applications, we then explored the use of this on‐demand click immobilization process to adjust hydrogel composition and control cell adhesion over time. 3D bioprinted constructs (squares of 5 mm × 5 mm with 3 layers) containing ASCs were incubated in culture medium in the absence of adhesive molecules. RGD‐N_3_ was then added to the culture medium to react with the polymer network and trigger cell adhesion, either 1 or 2 days after printing. Cell adhesion was assessed by monitoring cell sphericity, expecting a lower sphericity for adhering cells. In the absence of RGD‐N_3_, ASCs maintained their rounded shape, with a constant high sphericity of ≈0.90 throughout the 3‐day experiment (Figure 7a). Conversely, the sphericity of encapsulated ASCs exposed to immobilized RGD systematically decreased to ≈0.75 within 24 h following the RGD click immobilization, confirming cell adhesion. More importantly, cell adhesion could be triggered at different times (day 1 or day 2 after printing), demonstrating a temporal control over cell behavior after bioprinting (Figure S16, Supporting Information).

Lastly, we investigated the spatial control of post‐printing modifications. More precisely, by patterning clickable and non‐clickable dynamic hydrogels within a single construct, we set out to modify the local composition of a 3D‐printed object upon adding an N_3_‐bearing molecule to the culture medium. Squares (5 mm × 5 mm with 3 layers) of clickable dynamic hydrogels were 3D printed side‐by‐side with non‐clickable dynamic hydrogels and incubated together in PBS containing fluorescently labeled HA‐N_3_. After 24 h of incubation followed by washes of the unbound HA‐N_3_, the remaining fluorescence signal was only found in the clickable zones of the 3D‐printed object with a homogeneous distribution (Figure 7b), confirming the localized modification of the composition.

Overall, clickable dynamic bioinks constitute a promising approach to control the mechanical and biochemical properties of 3D bioprinted constructs over time and space. Though 4D printing has recently been explored for non‐biological objects,^[^ ^43^, ^44^ ^]^ with post‐printing adjustments of their shape based on salt addition, temperature change, or magnetic field,^[^ ^45^, ^46^ ^]^ to our knowledge, this study constitutes the first demonstration of the spatial and temporal control of the composition and mechanical properties of 3D bioprinted constructs under physiological conditions, offering a new way to control cell‐material interactions and cell behavior.

## 3 Conclusion

In this study, we introduced the new concept of clickable dynamic bioinks. Our results confirmed that optimized boronate ester‐based dynamic hydrogels are printable and that their constructs can withstand physiological conditions without support baths and/or complementary crosslinking. We showed that these hydrogels and their associated printing conditions are cytocompatible, meaning that they can be used in 3D bioprinting. Furthermore, chemically modifying our dynamic bioinks with click reactive moieties allowed us to fine‐tune the physical and biochemical properties of printed constructs with a variety of post‐printing modifications to adjust composition, stiffness, and cell adhesion properties, facilitating better control over cell fate. Markedly, we were also able to control these post‐printing modifications over time and space. In the near future, clickable dynamic bioinks could potentially be developed with any combination of dynamic hydrogel networks and click reactions, making clickable dynamic bioinks a universal concept for 4D bioprinting.

## 4 Experimental Section

### Materials

Sodium hyaluronate was purchased from Lifecore Biomedical (USA). Chondroitin sulfate 6 sodium salt (CS) was purchased from Carbosynth (USA). Alginate (Protanal LF10/60) was purchased from FMC Corporation (USA). 2‐(N‐morpholino)ethanesulfonic acid (MES), 4‐(4,6‐dimethoxy‐1,3,5‐triazin‐2‐yl)−4‐methylmorpholinium chloride (DMT‐MM), and D‐glucamine were purchased from TCI Chemicals (Belgium). Azido‐PEG2‐amine was purchased from Broadpharm (USA). Wulff's type phenylboronic acid (wPBA) was kindly provided by the CEISAM laboratory (Nantes, France). Dulbecco's Phosphate Buffered Saline (without Ca & Mg), Dulbecco's Modified Eagle Medium (DMEM), Penicillin/Streptomycin (P/S), Alexa Fluor 568, Alexa Fluor 488, phalloidin, Hoechst 33 258, actinomycin‐D, CCK‐8, PicoGreen Reagent, and the Live/Dead Kit were purchased from Thermo Fischer Scientific. Fetal Bovine serum (FBS) was purchased from Dominique Dutscher. Otherwise stated, all other reagents were purchased from Sigma‐Aldrich.

### Polysaccharide Modifications—Synthesis of HA‐wPBA

HA‐wPBA was synthesized as previously described with minor modifications.^[^ ^30^ ^]^ Briefly, 100 mg of sodium hyaluronate (HA) of 100 or 200 kDa) were dissolved at 10 mg.mL^−1^ in MES buffer (pH = 5.5), under stirring and at room temperature. After complete dissolution, DMT‐MM (137.9 mg [2 eq] for HA 100‐kDa HA; 69 mg [1 eq] for 200‐kDa HA) was added and allowed to react for 30 min. wPBA (55.3 mg [1 eq] for 100‐kDa HA; 27.7 mg [0.5 eq] for 200‐kDa HA) was added and allowed to react for 3 days at room temperature while stirring. The solutions containing the chemically modified polymers were dialyzed against 0.1 M PBS for 24 h, then against deionized water for 48 h, before filter sterilization, lyophilization, and storage at −20 °C. The degree of substitution of HA‐wPBA was determined by ^1^H NMR (400 MHz, D_2_O): the N‐acetyl group of HA (3 protons at 2.1 ppm) served as a reference to quantify the relative amount of grafted wPBA (4 protons at 7.5‐8 ppm), as previously reported.^[^ ^30^ ^]^

### Polysaccharide Modifications—Synthesis of HA‐Glucamine

HA‐glucamine was synthesized as previously described with minor modifications.^[^ ^30^ ^]^ Briefly, 100 mg of HA (100 or 200 kDa) was dissolved at 10 mg.mL^−1^ in MES buffer (pH = 5.5) at room temperature while stirring. After complete dissolution, DMT‐MM (2 eq, 137.9 mg) was added and allowed to react for 30 min. Glucamine (1 eq, 45.1 mg) was then added and allowed to react for 3 days at room temperature while stirring. The solutions containing the chemically modified polymers were dialyzed against 0.1 M PBS for 24 h, then against deionized water for 48 h, before filter sterilization, lyophilization, and storage at −20 °C. The degree of substitution of HA‐glucamine was determined by dosing the unreacted glucamine from the reaction medium using a 5% solution of TNBSA (2,4,6‐Trinitrobenzenesulfonic acid), according to the manufacturer's recommendations.

### Polysaccharide Modifications—Synthesis of BCN‐Modified HA‐Glucamine

100 mg of HA‐glucamine was dissolved in 9 mL of MES buffer (pH = 5.5) at room temperature while stirring. After complete dissolution, DMT‐MM (0.4 eq, 23.1 mg) was added and allowed to react for 30 min. N‐[(1R,8S,9s)‐Bicyclo[6.1.0]non‐4‐yn‐9‐ylmethyloxycarbonyl]−1,8‐diamino‐3,6‐dioxaoctane (BCN, 0.2 eq, 13.5 mg) was dissolved in 1 mL DMSO, added to the solution of HA‐glucamine, and allowed to react for 3 days at room temperature while stirring. The solution containing the chemically modified polymers were dialyzed against 0.1 M DPBS containing 2% DMSO overnight, 0.1 M DPBS for 8 h, and deionized water for 2 days, prior to filter sterilization, lyophilization, and storage at −80 °C. The degree of substitution of BCN‐modified HA‐glucamine was determined by ^1^H NMR (400 MHz, D_2_O): the N‐acetyl group of HA (3 protons, 2.1 ppm) served as a reference to quantify the relative amount of grafted BCN (2 protons at 1.1 ppm, 2 protons at 2.4 ppm, and 2 protons at 4.3 ppm).

### Polysaccharide Modifications—Synthesis of HA‐N_3_ and CS‐N_3_: N_3_

Modified polysaccharides were synthesized as previously described with minor modifications.^[^ ^31^ ^]^ Briefly, 100 mg of HA (20 kDa) or CS was dissolved at 10 mg.mL^−1^ in MES buffer (pH = 5.5) at room temperature while stirring. After complete dissolution, DMT‐MM (69 mg [1 eq] for HA; 60.2 mg [1 eq] for CS) was added and allowed to react for 30 min. Then, azido‐PEG2‐amine (21.7 mg [0.5 eq] for HA; 0.5 eq, 19 mg [0.5 eq]for CS) was added and allowed to react for 3 days at room temperature while stirring. The solutions containing the chemically modified polymers were dialyzed against 0.1 M PBS for 24 h, then against deionized water for 48 hours, before filter sterilization, lyophilization, and storage at −20 °C. The degrees of substitution of HA‐N_3_ and CS‐N_3_ were determined by ^1^H NMR (400 MHz, D_2_O): the N‐acetyl group of HA or CS (3 protons at 2.1 ppm) served as a reference to quantify the relative amount of grafted N_3_ (13 protons between 3.3 and 4.1 ppm), as previously reported.^[^ ^31^ ^]^

### Polysaccharide Modifications—Synthesis of Gelatin‐N_3_

50 mg of gelatin was dissolved at 10 mg.mL^−1^ in MES buffer (pH = 5.5) at room temperature while stirring. After complete dissolution, 10 mg of DMT‐MM was added and allowed to react for 30 min. Then, 35 mg of azido‐PEG2‐amine was added and allowed to react for 3 days at room temperature while stirring. The solutions containing the chemically modified polymers were dialyzed against 0.1 M PBS for 24 h, then against deionized water for 48 h, before filter sterilization, lyophilization, and storage at −20 °C.

### Polysaccharide Modifications—Synthesis of Fluorescent Polymers

Fluorescent polymers were obtained following a previous protocol with minor modifications.^[^ ^31^ ^]^ Briefly, Either HA, HA‐wPBA, HA‐N_3_, CS, or CS‐N_3_ (50 mg) was dissolved at 10 mg.mL^−1^ in MES buffer (pH = 5.5) at room temperature while stirring. Some 3 mg of DMT‐MM was added and allowed to react for 30 min. Then, 0.5 mg of a hydrazide‐modified fluorophore (CF488 or CF647) was dissolved in 0.5 mL of DMSO and added to the polymer solution and allowed to react in the dark for 3 days at room temperature while stirring. The solutions containing the chemically modified polymers were dialyzed against 0.1 M PBS for 24 h, then against deionized water for 48 h, before filter sterilization, lyophilization, and storage at −20 °C.

### Cell Isolation and Culture

Primary human cells were isolated from various tissues with donor agreement. Human adipose‐derived stromal cells (ASCs) were isolated from lipoaspirates as previously described,^[^ ^47^ ^]^ seeded at 10000 cm^−^^2^, and amplified in growth medium (Promocell, Heidelberg, Germany) with 1% P/S and 1% amphotericin B (ATB), changing medium every other day. ASCs were used between passages 2 and 5. Chondrocytes were isolated from surgical wastes of non‐osteoarthritic vertebral transverse costal facet joints from patients undergoing scoliosis surgery (HC, 15–21 years old) or from osteoarthritic knees of patients undergoing a total knee surgery (OAC,71–81 years old). According to the Declaration of Helsinki, all human tissue samples were harvested from patients after their informed consent. This study was carried out in accordance with the recommendation of the “Comité pour la Protection des Personnes” of Pays de La Loire and approved by the French Ministry of Higher Education and Research (registration number: DC‐2017‐2987). Cartilaginous tissues were sliced in chips before matrix degradation successively with hyaluronidase (1 mg.mL^−1^, 10 min at 37 °C; 750–1500 U.mg^−1^), trypsine (8 µg.mL^−1^, 15 min at 37 °C; 12100 U.mg^−1^), and collagenase (1.2 mg.mL^−1^, 14 h at 37 °C; 452 U.mg^−1^). Then, the digested tissues were filtered on a 70‐µm cell strainer before filtrate centrifugation (260 g, 3 min). The isolated cells were then suspended in complete culture medium (DMEM 10% FCS, 1% P/S, 1% ATB) and seeded at 10000 cm^−2^ for amplification in 75‐cm^2^ culture flasks, changing medium every other day. Primary chondrocytes were used at passage 0 to 1.^[^ ^47^, ^48^ ^]^

### Preparation of Hydrogels and Bioinks

Hydrogels were obtained by separately dissolving HA‐wPBA, HA‐glucamine, and/or BCN‐modified HA‐glucamine in PBS prior to mixing them together in a chosen volume ratio. Dynamic hydrogels were formed by mixing HA‐wPBA and HA‐glucamine.

One formulation was composed of 1% (w/v) of HA (200 kDa), combining HA‐wPBA (DS of 24%) and HA‐glucamine (DS of 52%) with a wPBA:glucamine molar ratio of 1:1; the other formulation is composed of 2.5% (w/v) of HA (100 kDa), combining HA‐wPBA (DS of 40%), and HA‐glucamine (DS of 52%) with a wPBA:glucamine molar ratio of 1:1. Clickable dynamic hydrogels were formed by mixing HA‐wPBA and BCN‐modified HA‐glucamine in the wPBA:glucamine molar ratio of 1:1. To obtain bioinks, cells were resuspended in PBS containing HA‐glucamine (or BCN‐modified HA‐glucamine) prior to mixing with HA‐wPBA. All of the experiments were conducted with a final concentration of 1 × 10^6^ cells per mL of hydrogel.

### Hydrogel Rheological Characterization

Rheological characterization was performed using a HAAKE™ MARS™ (Thermo Fischer Scientific, France) equipped with a 20 mm‐wide titanium upper plate and a Peltier plate to control the temperature (200‐µL samples). Hydrogel formation was assessed by oscillatory time sweep experiments (1 Pa, 1 Hz) performed within the linear viscoelastic region. Gelation time was determined as the time when the shear storage modulus (Gʹ) value equaled that of the shear loss modulus (G″). The dynamic behavior of the hydrogels was characterized via oscillatory frequency sweep experiments (0.01 to 1 Hz) under a stress of 1 Pa. The shear‐thinning property of the hydrogels was characterized via rotation ramp experiments (1 Pa) by applying shear rates (ɣ̇) of 0.01 to 1000 s^−1^. The flow points of dynamic hydrogels were assessed via stress sweep experiments (1 Hz) over a range of shear stress (τ) of 0.1 to 1000 Pa. The self‐healing properties of the hydrogels were evaluated by alternating the application of low (1 Pa, at 1 Hz for 120 s) and high (500 or 2000 Pa, 1 Hz for 30 s) stress.

### Hydrogel Stiffness Evaluation

Hydrogels were cast between two glass slides separated by a 1 mm‐thick silicon joint. After 1 h, the hydrogels (150 µL) were immersed in PBS (500 µL) and incubated overnight at 37 °C. The hydrogels were then punched (2 mm in diameter) and subjected to unconfined 20% strain compression tests using a Microtester (CellScale, Canada). Young's modulus (E) was calculated with the following equation:

(1) $$E=stress/strain=\left(F/A\right)/\left(\Delta I/I_{0}\right)$$

where F is the force applied to the hydrogel (N); A, the area onto which the force is applied (m^2^); ΔI, the height displacement (µm); and I_0_, the initial thickness (µm). For each formulation, three distinct hydrogels were formed and punched in at least 4 locations.

### 2D and 3D Printing Experiments

An RGen‐200 bioprinter (RegenHU, Villaz‐St‐Pierre, Switzerland) placed in a biosafety cabinet was used for all of the 3D bioprinting experiments. The G‐code used for the operation of the printer was generated with the Shaper software (RegenHU). The hydrogels were formed in 2 mL microcentrifuge tubes for homogeneous mixing, then transferred to a sterile 3 mL bioprinting cartridge (Nordson, Westlake, USA) and connected to the bioprinter. All printed constructions were obtained via pneumatic extrusion bioprinting using a 22 G nozzle at room temperature. Optimal printing conditions were determined by systematically screening combinations of input air pressures and printing head displacement speeds for each hydrogel formulation. For printability assessment, the surface of printed strands over a fixed length of 1 cm was characterized. To do so, pictures of the printed filaments from above was first taken. Then, the area of 1‐cm long portions of filaments was measured using the Fiji software by applying the “Analyze particle” tool to manually outlined surfaces.

### Stability of 3D‐Printed Constructs

Hydrogel cylinders (6 mm wide with 10 layers) were 3D printed as described above and placed on 3D‐printed polylactic acid (PLA) disks (DiscoEasy200) for easy handling. The 3D‐printed hydrogels were then placed in culture medium. To assess the stability of printed constructs, height was measured daily with an electronic digital caliper (Thermo Fisher Scientific) and normalized to the initial values (D0).

### Basic Biological Function Assessment

Cell viability, metabolic activity, and DNA content were assessed at day 0, 3, and 7 using LIVE/DEAD™ staining, the CCK‐8 assay, and the PicoGreen assay, respectively. For LIVE/DEAD™ staining, hydrogels were transferred to an 8‐well chamber slide (IBIDI) and incubated with calcein AM (living cells) and ethidium homodimer (dead cells) per the manufacturer's recommendations. Images were acquired via confocal microscopy (A1RS, Nikon). Cell viability was calculated as the number of living cells divided by the total number of cells. Regarding the metabolic activity assay, hydrogels were transferred to a 48‐well plate and incubated for 2 h with a CCK‐8 solution per the manufacturer's recommendations. The absorbance of the supernatant at 460 nm was measured (Tristar 2S plate reader, Berthold Technologies), and the results were normalized to D0. Regarding the evaluation of DNA content, hydrogels were transferred to a 48‐well plate and washed with PBS. After PBS removal, 250 µL of TE 1X was added on each hydrogel, which was then placed at −80 °C for 24 h. The DNA quantification was subsequently performed per the manufacturer's instructions.

### Cell Sedimentation

Cell sedimentation in dynamic bioinks was assessed at 10 min and 4 h after cell encapsulation (10^6^ cells per mL of bioink) and compared to that in viscous solutions of unmodified HA with similar molecular weight (200 or 100 kDa) and polymer content (1% or 2.5% [w/v]). Cells were visualized using LIVE/DEAD™ staining and confocal imaging (A1RS, Nikon, France), and calcein AM (living cells) and ethidium homodimer (dead cells) staining was performed per the manufacturer's recommendations. Cell distribution was assessed along the z‐axis and expressed as the number of cells per 100‐µm section normalized to the total cell number.

### Diffusion and Immobilization of Clickable Macromolecules

Either fluorescently labeled (CF647) HA (20 kDa), HA‐N_3_ (20 kDa), CS, or CS‐N_3_ was dissolved in PBS at 1 mg.mL^−1^. Then, 150 µL of the fluorescent polymer solution was added on top of clickable dynamic hydrogels (50 µL) previously printed in 16‐well chamber slide. The diffusion of fluorescent polymers throughout the hydrogels was evaluated by monitoring the fluorescence for up to 24 h at 37 °C using confocal imaging. To demonstrate the polymer immobilization, hydrogels were washed with PBS every 24 h over 3 days at 37 °C before fluorescence assessment using confocal microscopy.

### Immobilization of Clickable Adhesive Molecules

Clickable dynamic hydrogels were formed and 3D printed as aforementioned. N_3_‐modified adhesive macromolecules (i.e., N_3_‐KGSGSGRGDSP and gelatin‐N_3_) were dissolved in culture medium (concentrations of 300 µM and 10 mg mL^−1^, respectively) and added on top of clickable dynamic hydrogels before 24‐hour incubation (37 °C, 5% CO_2_, and humid atmosphere). To assess the ability of cells to adhere onto the modified clickable dynamic hydrogels, the hydrogels were washed 3 times with PBS before seeding ASCs at 2.10^4^ cells.cm^−2^ onto the hydrogels and allowing them to adhere for 24 h. Hydrogels were then washed 3 times with PBS to remove non‐adhering cells. The remaining cells were fixed with 4% paraformaldehyde (15 min at room temperature), permeabilized with 0.1% Triton X‐100 (30 min at room temperature), and labeled with both Alexa Fluor 568 phalloidin (1/200^e^, 1 hour at room temperature) to visualize F‐actin (expressed as actin area per surface unit), and then Hoechst 33 258 (1/50000^e^, 30 min at room temperature) to visualize nuclei. Images were acquired with an AxioZoom Macroscope and analyzed with the Fiji software for fluorescence quantification. Regarding nuclei quantification, the number of nuclei (Hoechst staining, in blue) was first determined using the “Analyze Particles” tool. The nuclei density (in nuclei cm^−2^) was then calculated by dividing the number of nuclei by the total surface of an acquired image. Regarding F‐actin quantification, the area of F‐actin (Alexa Fluor 568 phalloidin staining, in red) was determined using the “Analyze Particles” tool. The F‐actin area was then expressed as actin area per surface unit (in µm^2^ cm^−2^) by dividing the F‐actin area by the total surface of an acquired image.

### Spatiotemporal Control of Post‐Printing Bioink Modifications—Assessment of Temporal Control

Hydrogels were extruded (5 mm disks with 3 layers) in 12‐well plate culture dishes, then incubated in PBS for 0, 3, or 7 days before a 24‐hour incubation in PBS containing fluorescently labeled (CF647) HA‐N_3_ (20 kDa, 1 mg.mL^−1^). After washes with PBS, images were acquired with an AxioZoom Macroscope and treated with the Fiji software for fluorescence quantification.

### Spatiotemporal Control of Post‐Printing Bioink Modifications—Assessment of Spatial Control

Clickable and non‐clickable dynamic hydrogel squares (5 mm wide squares with 3 layers) were 3D printed side‐by‐side in a 12‐well plate culture dish and incubated for 24 h in PBS containing fluorescently labeled (CF647) HA‐N_3_ (20 kDa, 1 mg.mL^−1^). After washes with PBS, images were acquired with an AxioZoom Macroscope and treated with the Fiji software for fluorescence quantification.

### Spatiotemporal Control of Post‐Printing Bioink Modifications—Temporal Control Over Cell Adhesion

36 squares (3 layers) of soft clickable dynamic bioink containing ASCs were 3D bioprinted in 8‐well microscopy glass slides, and immersed in complete culture medium prior to incubation. On day 1 or day 2 after printing, the culture medium was replaced be culture medium containing 300 µM RGD‐N_3_. Cell adhesion was evaluated by measuring cell sphericity on day 0, 1, 2, and 3. To do so, cells within the 3D bioprinted constructs were fixed with 4% PFA (30 min at room temperature), permeabilized with 0.1% Triton X‐100 (10 min at room temperature), and successively labeled with Alexa Fluor 568 phalloidin (1/200^e^, 1 h at room temperature) to visualize F‐actin, and Hoechst 33 258 (1/50000^e^, 30 min at room temperature) to visualize nuclei. Cells were imaged using confocal microscopy. Cell sphericity was calculated in three dimensions (voxel size = 0.178 µm^3^) using the 3D measurement sphericity of the NIS‐Elements AR Analysis 5.42.01 software with the following equation: $\mathrm{Sphericity}=\frac{\sqrt[{3}]{\pi {\left(6V\right)}^{2}}}{S}$, where *V* is the volume and *S* the surface.

### Statistical Analyses

All data are shown as mean ± standard deviation. Statistical analysis was carried out using GraphPad to evaluate statistical significance via Student's t‐test, one‐way ANOVA with Dunnett's post‐hoc test, or two‐way ANOVA with Tukey's post‐hoc test.

## Conflict of Interest

The authors declare no conflict of interest.

## Author Contributions

The following author contributions were identified based on the guidelines from CRediT (Contributor Roles Taxonomy). PT contributed to the conceptualization, methodology, investigation, and writing. VD contributed to the conceptualization, methodology, supervision, project administration, and writing. GSP and NL contributed to the investigation, the conceptualization, and the methodology. BH, FL and AT contributed to the resources. JG and CLV contributed to the writing (review and editing).

## Supporting information

Supporting Information

Supplemental Video 1

Supplemental Video 2

## Data Availability

The data that support the findings of this study are available from the corresponding author upon reasonable request.
