# Supplementary material for: Clickable Dynamic Bioinks Enable Post‐Printing Modifications of Construct Composition and Mechanical Properties Controlled over Time and Space
Source: Adv Sci (Weinh). 2023 Sep 15;10(30):2300055. doi: 10.1002/advs.202300055 (PMC10602521; doi:10.1002/advs.202300055)
Supplement: Supplementary file 1 — Supporting Information [file ADVS-10-2300055-s001.pdf]

ADVANCED SCIENCE
Open Access

Supporting Information

for *Adv. Sci.*, DOI 10.1002/advs.202300055

Clickable Dynamic Bioinks Enable Post-Printing Modifications of Construct Composition and Mechanical Properties Controlled over Time and Space

*Pierre Tournier, Garance Saint-Pé, Nathan Lagneau, François Loll, Boris Halgand, Arnaud Tessier, Jérôme Guicheux, Catherine Le Visage and Vianney Delplace**

Supporting Information

Clickable Dynamic Bioinks Enable Post-Printing Modifications of Construct Composition and Mechanical Properties Controlled over Time and Space

*Pierre Tournier, Nathan Lagneau, Boris Halgand, François Loll, Arnaud Tessier, Jérôme Guicheux, Catherine Le Visage, and Vianney Delplace**

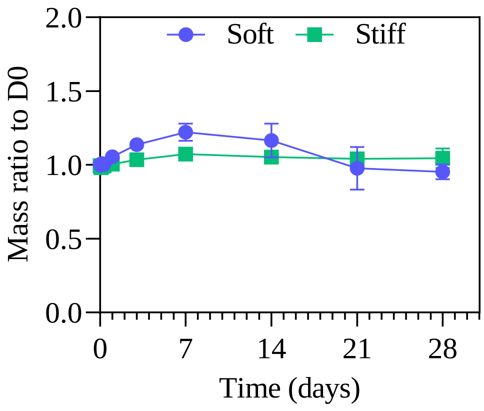

Fig. S1. Swelling/stability study of the soft and stiff dynamic hydrogels in PBS at 37°C.

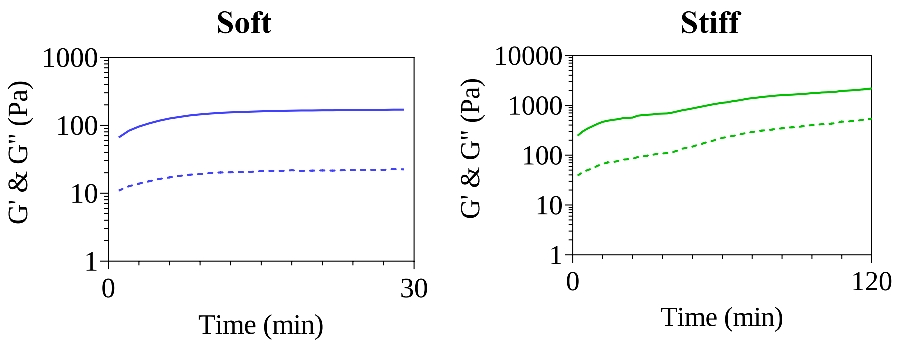

Fig. S2. Shear storage moduli (G′, continuous line) and shear loss moduli (G″, dotted line) of the soft and stiff dynamic hydrogels, obtained via dynamic shear rheometry.

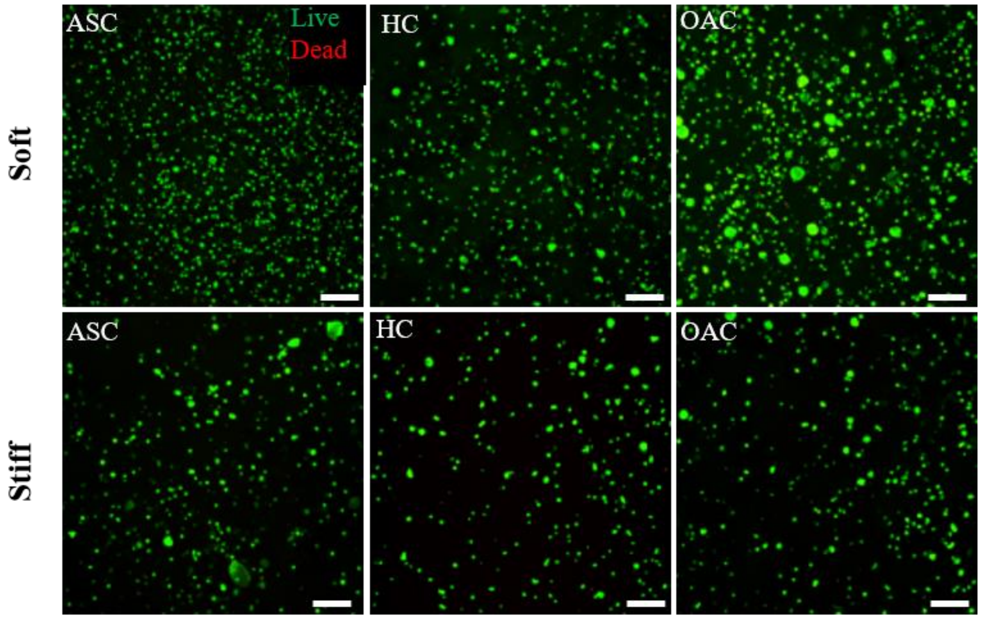

Fig. S3. Cytocompatibility of the soft and stiff hydrogels in combination with the bioprinting process. After encapsulation and extrusion, the viability of three cell types (primary human adipose-derived stromal cells [ASCs], chondrocytes from healthy vertebrae [HC], and chondrocytes from osteoarthritis knees [OACs]) was assessed using LIVE/DEAD™ staining. Representative stack views are provided (scale bar = 250 µm).

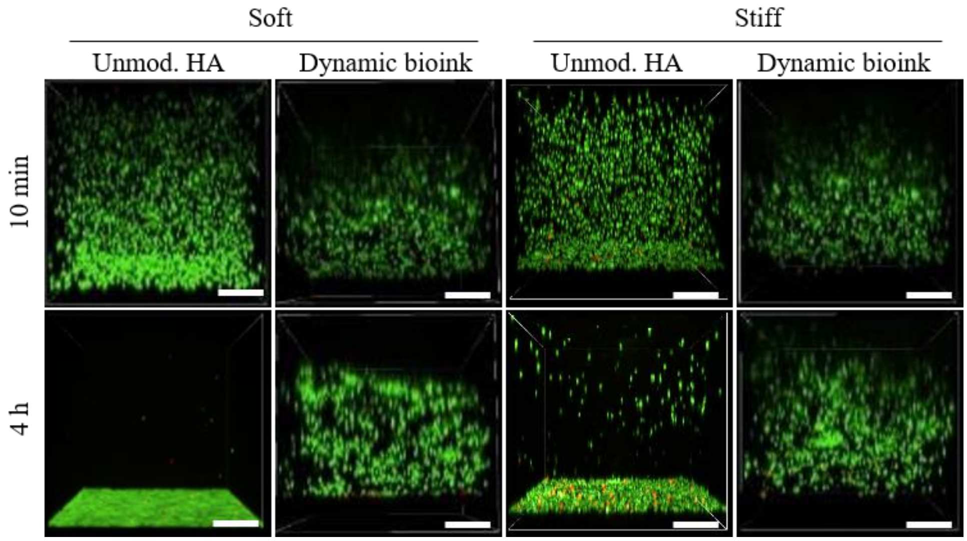

Fig. S4. 3D views of the distribution of primary human adipose-derived stromal cells (ASCs) encapsulated in the soft and stiff dynamic hydrogels or resuspended in an unmodified HA solution. Comparison of cell distribution 10 min vs 4 hours after encapsulation demonstrated the absence of cell sedimentation only for the dynamic bioinks. (Scale bar = 250 µm).

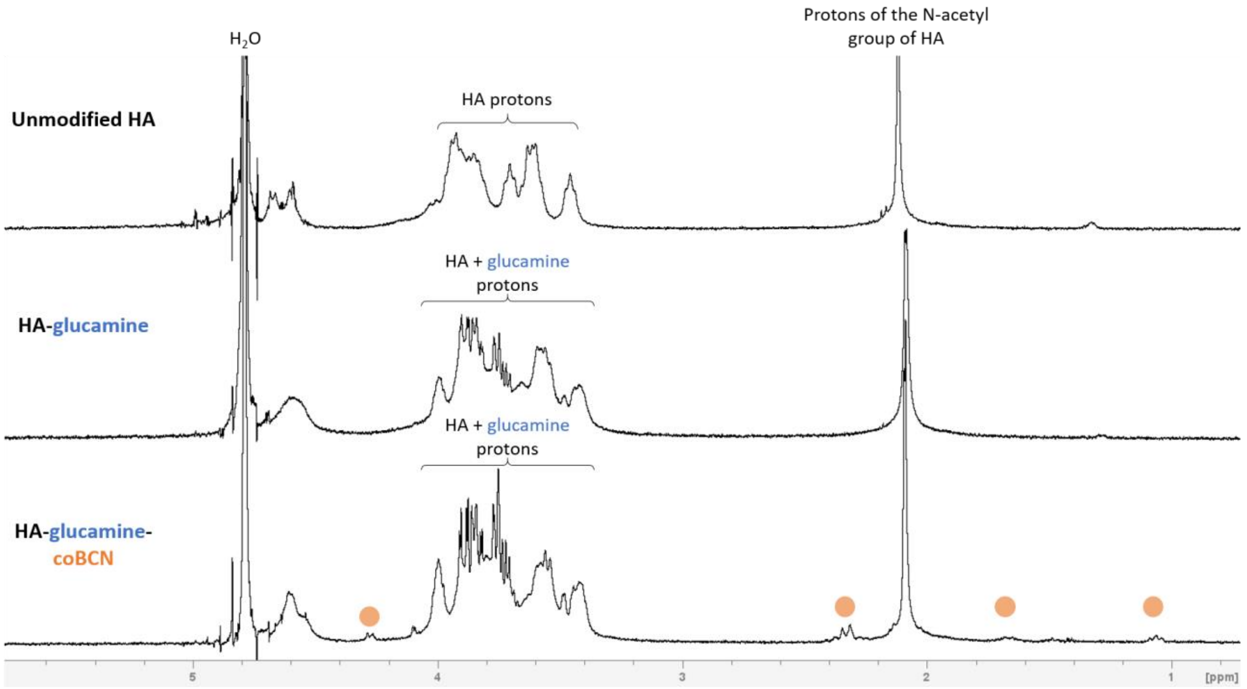

Fig. S5. Representative ^{1}H NMR (400 MHz, D_2O) of unmodified HA, HA-glucamine, and BCN-modified HA-glucamine, confirming the successful chemical modification of HA. The 3 protons of the N-acetyl group of HA (2.1 ppm) served as a reference to calculate the degrees of BCN substitution of BCN-modified HA-glucamine (orange circles, including 2 protons at 1.1 ppm, 2 protons at 2.4 ppm, and 2 protons at 4.3 ppm).

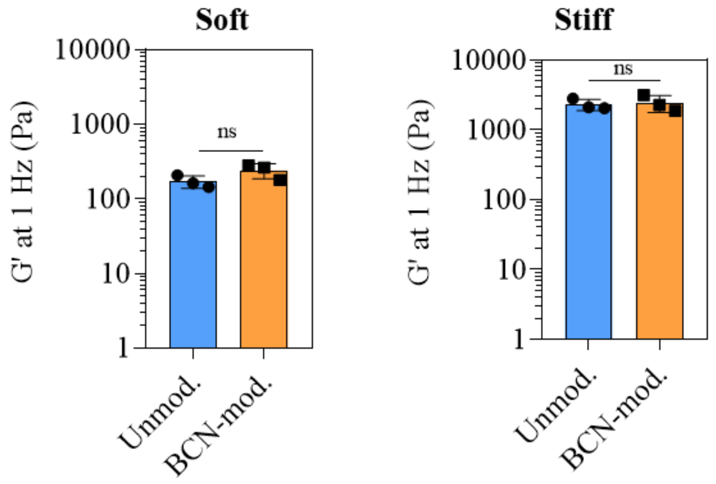

Fig. S6. Shear storage moduli (G′, at 1 Hz) of the soft (left) and stiff (right) dynamic hydrogels either unmodified (unmod.) or modified with BCN for click reaction (BCN-mod.). Values were extracted from frequency sweep experiments performed at 37 °C with a dynamic shear rheometer. Data are shown as mean ± SD (n = 3) with statistical significance determined using Student's t-test (ns: not significant).

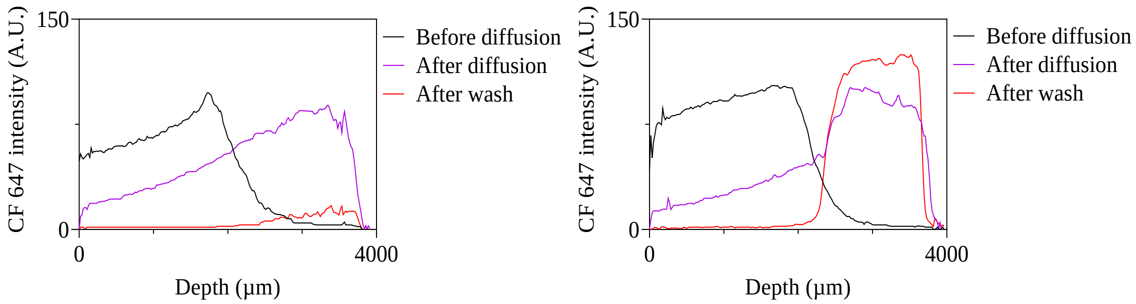

Fig. S7. Quantification of the CF 647 HA (left) and CF647 HA-N_3 (right) fluorescence intensity in the soft clickable dynamic hydrogel.

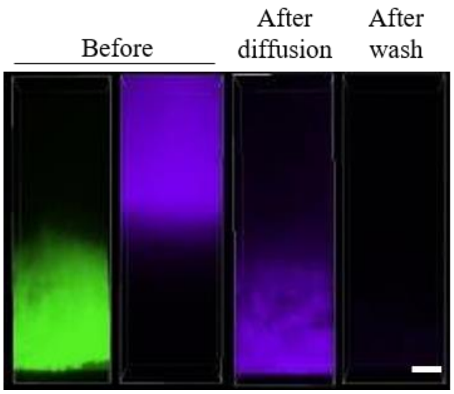

Fig. S8. The incubation of an unmodified soft dynamic hydrogel (green fluorescence) with fluorescent HA-N_3 (purple fluorescence) did not allow for subsequent polymer immobilization. After successful diffusion within the hydrogel, the fluorescence from HA-N_3 disappears with washes, indicating the absence of HA-N_3 immobilization within the non-clickable dynamic hydrogel (scale bar = 500 μm).

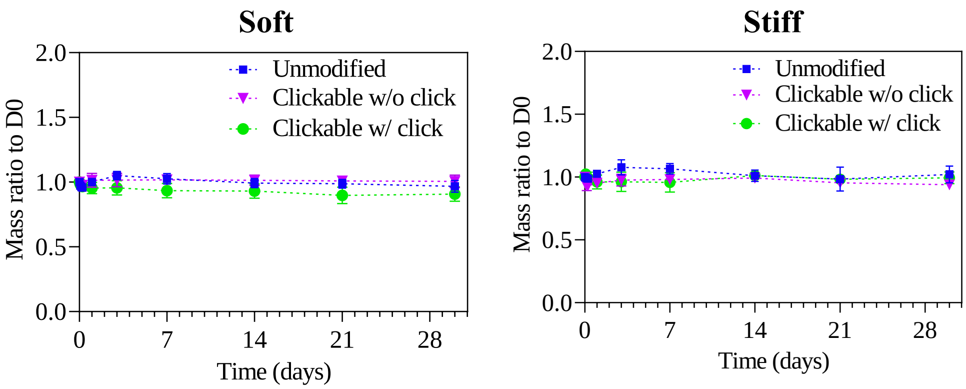

Fig. S9. Stability of the soft (left) and stiff (right) dynamic hydrogels in culture medium. Clickable dynamic hydrogels were prepared in PBS then immersed in DMEM with or without HA-N_3 (0.05% w/v for the soft and 0.125% w/v for the stiff formulations) for up to 30 days. Similarly to unmodified dynamic hydrogels, the clickable dynamic hydrogels showed minimal swelling/shrinking, confirming that the clickable moieties and the click reaction do not alter the hydrogel stability.

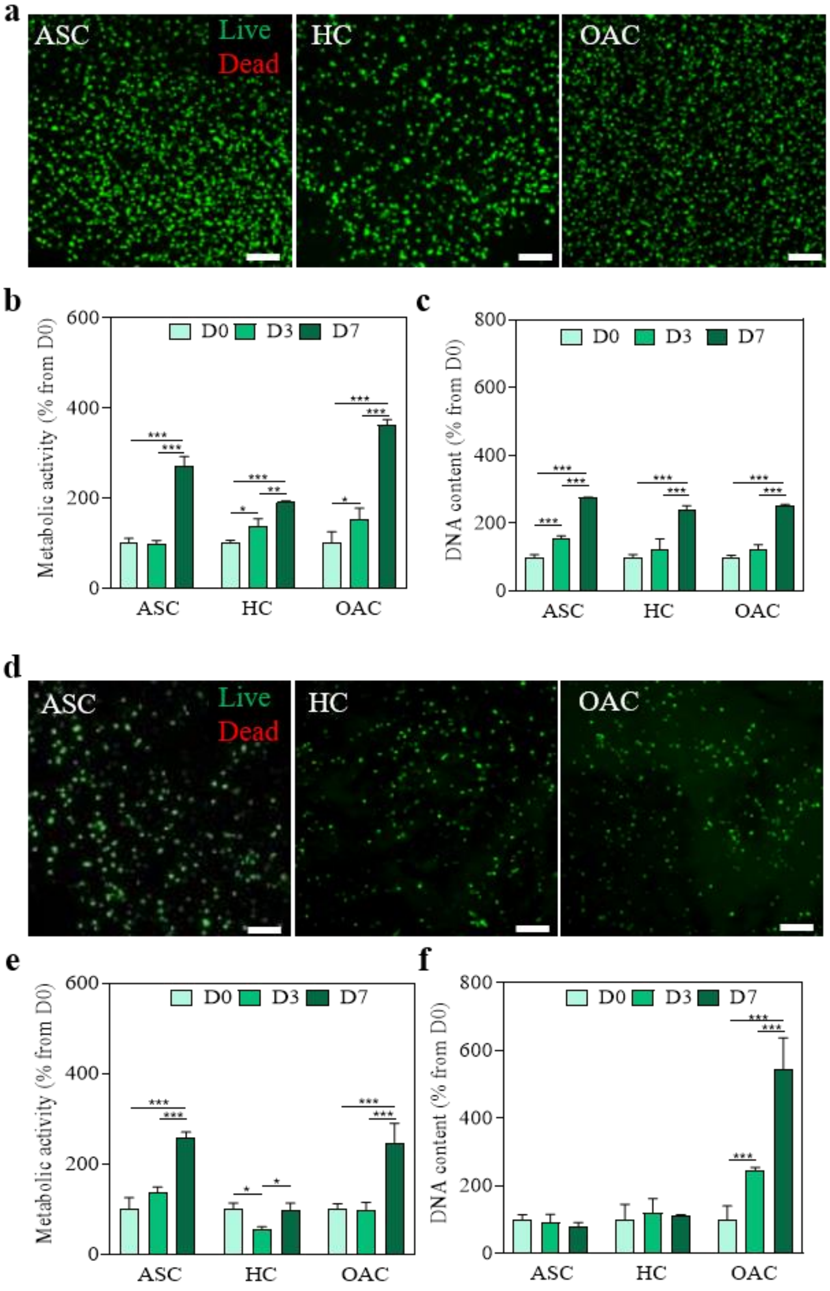

Fig. S10. Evaluation of the cytocompatibility of the soft (a-c) and stiff (d-f) clickable dynamic bioinks. The viability of encapsulated cells (three cell types: primary human adipose-derived stromal cells [ASCs], chondrocytes from healthy vertebrae [HC], and chondrocytes from osteoarthritis knees [OACs]) was assessed on day 0, 3, and 7 using three distinct methods: LIVE/DEAD™ staining, metabolic assay (CCK-8), and DNA quantification (PicoGreen) (scale bar = 150 µm). Data are shown as mean ± SD (n = 3) with statistical significance determined using one-way ANOVA with a Tukey's post-hoc test (ns: not significant, $*p < 0.05$, $**p < 0.01$, and $***p < 0.001$).

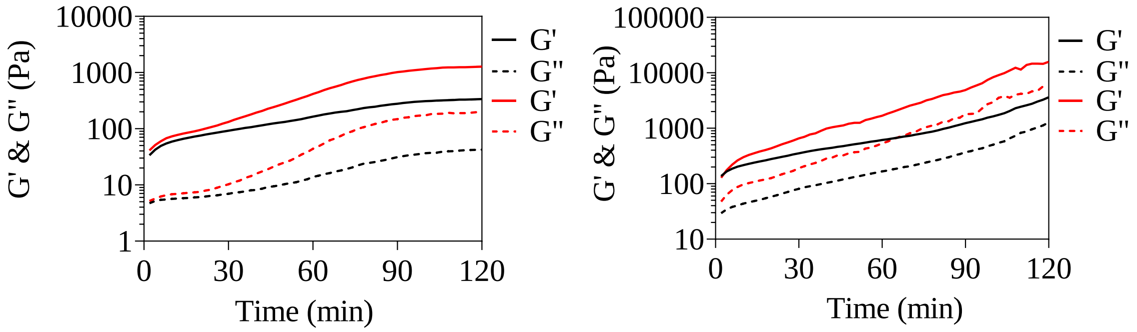

Fig. S11. Time sweep experiments showing the stiffening of the soft (left) and stiff (right) clickable dynamic hydrogels after mixing with HA-N_3 (N_3:BCN molar ratio of 1:1), via dynamic shear rheometry. The red and black lines indicate the clickable dynamic hydrogel with and without HA-N_3, respectively. The results show an important increase in shear moduli in the presence of HA-N_3, suggesting successful covalent crosslinking.

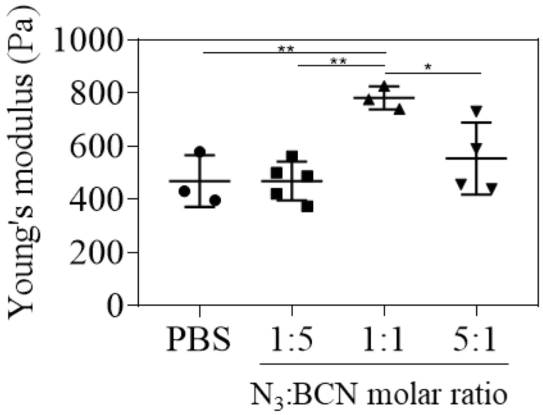

Fig. S12. Effect of the N_3:BCN molar ratio on the stiffening of clickable dynamic bioinks in the presence of HA-N_3. The variations in Young's modulus show that a significant increase in stiffness is obtained only with the N_3:BCN molar ratio of 1:1. Data are shown as mean ± SD (n = 3-5) with statistical significance determined using one-way ANOVA with a Tukey's post-hoc test (*$p < 0.05$, **$p < 0.01$).

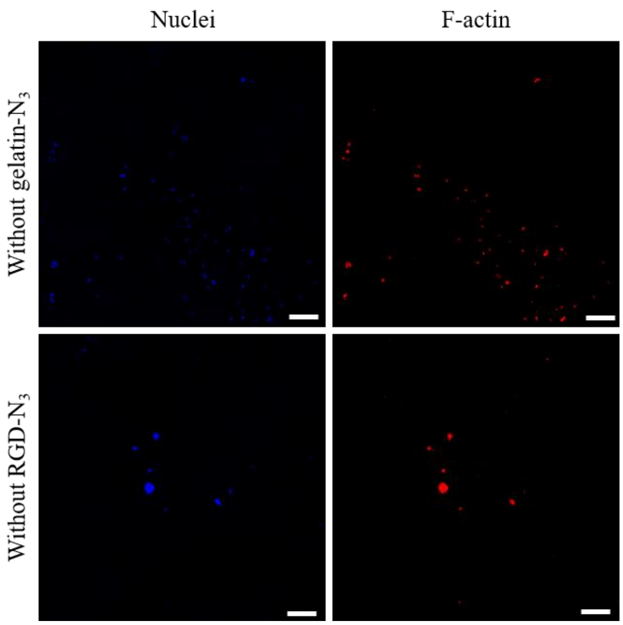

Fig. S13. Evaluation of the cell adhesion on the surface of clickable dynamic hydrogels in the absence of immobilized RGD-N_3 or gelatin-N_3. Cell adhesion was evaluated using Hoechst (nuclei, blue) and AF 568 phalloidin (F-actin, red) staining of primary human adipose-derived stromal cells (ASCs) after overnight seeding and several washes. Few cells were found compared to RGD- and gelatin-containing hydrogels, confirming the promotion of cell adhesion only in the presence of adhesive macromolecules (scale bar = 200 µm).

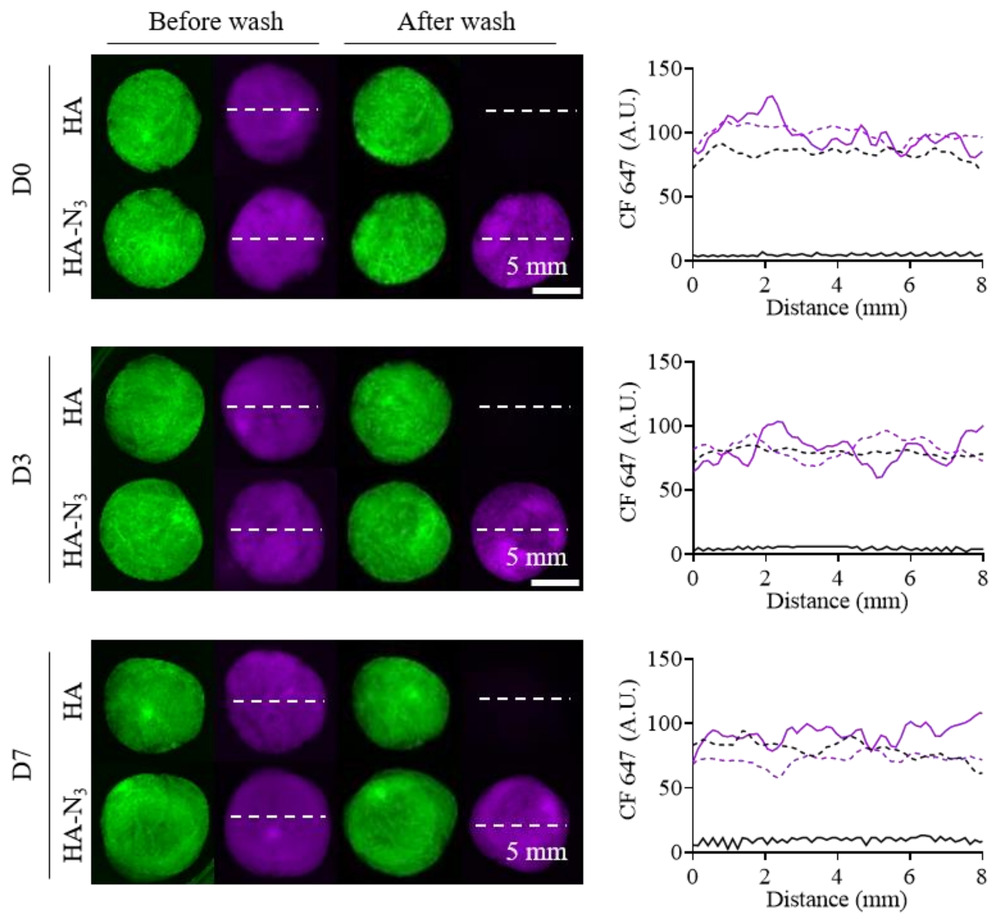

Fig. S14. Left: Top views of clickable dynamic hydrogels (CF488) incubated in CF647 HA (20 kDa) or CF647 HA-N_3 (20 kDa) up to 3 days post-printing. Fluorescence imaging was performed after 24 hours of incubation, and before or after washes. The CF647 signal was measured along the white dashed lines. Right: CF647 fluorescence intensity along the dashed white arrows, confirming local modification of clickable dynamic hydrogels.

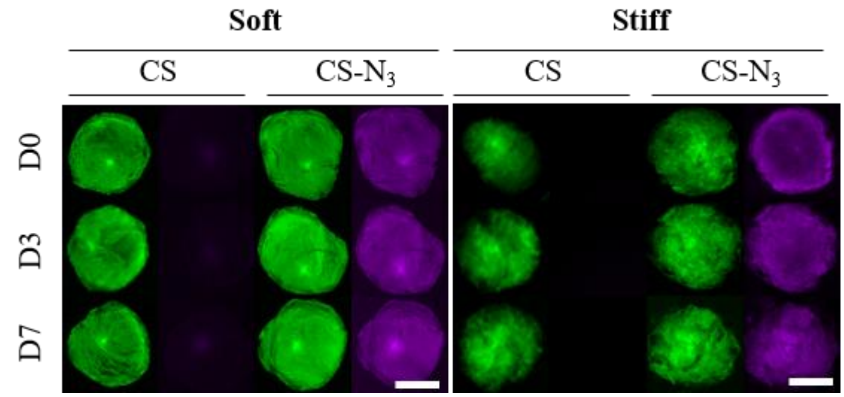

Fig. S15. Temporal control over the composition adjustment of clickable dynamic hydrogels with N_3-modified chondroitin sulfate (CS-N_3). Clickable dynamic hydrogels were incubated for 24 hours with a solution containing fluorescent CS or CS-N_3 (1 mg.mL^{-1}) on day 0, 3, or 7 after hydrogel preparation. After washes, only CS-N_3 remained in the hydrogel, suggesting specific immobilization of CS-N_3 via click chemistry. CS-N_3 immobilization was effective at the three investigated time points, confirming that the second click reaction can be performed at a chosen time (scale bar = 2.5 mm).

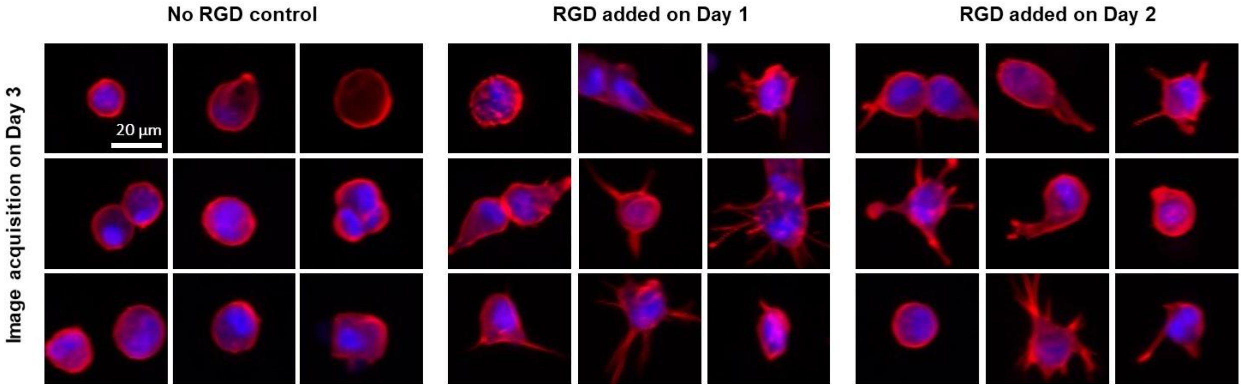

Fig. S16. Representative images of encapsulated ASCs exposed or not to clickable RGD immobilization. These images reveal a decrease in cell sphericity only upon the addition of the clickable adhesive peptide. Cell adhesion could be triggered at different times (day 1 or day 2 after printing), demonstrating a temporal control over cell behavior after bioprinting.

Supplementary Table 1. Synthetic conditions of the modified polymers used for the design of clickable dynamic hydrogels.

Target polymer	Polymer	Reaction medium	Activating agent (DMT-MM)	Grafted molecule	Substitution
200 kDa HA<br>wPBA 0.5 Eq	200 kDa HA;<br>100 mg (0.248 mmol)	MES 0.1 M,<br>pH=5.5 (9 mL)	69 mg (0.248 mmol)<br>1 Eq relative to carboxylic acid	wPBA in 1 mL MES buffer<br>28 mg (0.124 mmol)<br>0.5 eq relative to carboxylic acid	24% (^{1}H RMN)<br>20% (Elemental analysis)
200 kDa HA<br>Glucamine 1 Eq	200 kDa HA;<br>100 mg (0.248 mmol)	MES 0.1 M,<br>pH=5.5 (9 mL)	69 mg (0.248 mmol)<br>1 Eq relative to carboxylic acid	Glucamine in 1 mL MES buffer<br>45 mg (0.248 mmol)<br>1 eq relative to carboxylic acid	52 % (Elemental analysis)<br>56 % (TNBSA dosage)
200 kDa HA<br>Glucamine 1 Eq<br>BCN 0.2 Eq	200 kDa HA<br>Glucamine 1 Eq;<br>100 mg (0.211 mmol)	MES 0.1 M,<br>pH=5.5 (9 mL)	23 mg (0.084 mmol)<br>0.4 Eq relative to carboxylic acid	BCN in 1 mL DMSO<br>14 mg (0.042 mmol)<br>0.2 eq relative to carboxylic acid	6% (^{1}H RMN)
100 kDa HA<br>wPBA 1 Eq	100 kDa HA;<br>100 mg (0.248 mmol)	MES 0.1 M,<br>pH=5.5 (9 mL)	137 mg (0.496 mmol)<br>2 Eq relative to carboxylic acid	wPBA in 1 mL MES buffer<br>55 mg (0.248 mmol)<br>1 eq relative to carboxylic acid	40% (^{1}H RMN)
100 kDa HA<br>Glucamine 1 Eq	100 kDa HA;<br>100 mg (0.248 mmol)	MES 0.1 M,<br>pH=5.5 (9 mL)	137 mg (0.496 mmol)<br>2 Eq relative to carboxylic acid	Glucamine in 1 mL MES buffer<br>45 mg (0.248 mmol)<br>1 eq relative to carboxylic acid	52 % (Elemental analysis)<br>55 % (TNBSA dosage)
100 kDa HA<br>Glucamine 1 Eq<br>BCN 0.2 Eq	100 kDa HA<br>Glucamine 1 Eq;<br>100 mg (0.211 mmol)	MES 0.1 M,<br>pH=5.5 (9 mL)	23 mg (0.084 mmol)<br>0.4 Eq relative to carboxylic acid	BCN in 1 mL DMSO<br>14 mg (0.042 mmol)<br>0.2 eq relative to carboxylic acid	6% (^{1}H RMN)
20 kDa HA<br>N_3 0.5 Eq	20 kDa HA;<br>100 mg (0.248 mmol)	MES 0.1 M,<br>pH=5.5 (9 mL)	69 mg (0.248 mmol)<br>1 Eq relative to carboxylic acid	N_3 in 1 mL MES buffer<br>22 mg (0.124 mmol)<br>0.5 eq relative to carboxylic acid	40% (^{1}H RMN)
CS<br>N_3 0.5 Eq	CS;<br>100 mg (0.218 mmol)	MES 0.1 M,<br>pH=5.5 (9 mL)	60 mg (0.218 mmol)<br>1 Eq relative to carboxylic acid	N_3 in 1 mL MES buffer<br>19 mg (0.109 mmol)<br>0.5 eq relative to carboxylic acid	-
Gelatin<br>N_3	Gelatin;<br>50 mg	MES 0.1 M,<br>pH=5.5 (9 mL)	10 mg (0.036 mmol)	N_3 in 1 mL MES buffer<br>35 mg (0.201 mmol)	-

Supplementary Table 2. Composition of the dynamic and clickable dynamic hydrogels.

Hydrogel	Polymer content	HA-wPBA	HA-Glucamine	wPBA:Glucamine molar ratio
"Soft"	1% (w/v)	200 kDa HA<br>wPBA 24%	200 kDa HA<br>glucamine 52%	1:1
"Soft clickable"	1% (w/v)	200 kDa HA<br>wPBA 24%	200 kDa HA<br>glucamine 52%<br>BCN 6%	1:1
"Stiff"	2.5% (w/v)	100 kDa HA<br>wPBA 40%	100 kDa HA<br>glucamine 52%	1:1
"Stiff clickable"	2.5% (w/v)	100 kDa HA<br>wPBA 40%	100 kDa HA<br>glucamine 52%<br>BCN 6%	1:1
